# Barriers to Patient Portal Adoption Among a Bilingual Patient Population by Analysis of Survey Findings from English- and Spanish-Speaking Patients: Information Needs Study

**DOI:** 10.2196/66717

**Published:** 2025-07-28

**Authors:** Jiahua Yang, Michael Mackert, Daniela De Luca, Sophia Annette Dove

**Affiliations:** 1 Stan Richards School of Advertising and Public Relations, University of Texas at Austin, 300 W. Dean Keeton St., Austin, TX, 78712-1069, United States, 1 2106178014; 2 Dell Medical School, University of Texas at Austin, Austin, TX, United States; 3 Center for Health Communication, University of Texas at Austin, Austin, TX, United States

**Keywords:** patient portal adoption, patient portals, health communication, patient access to information, patient information, electronic health records, EHRs, electronic medical records, EMRs, patient record, health records, personal health record, PHR

## Abstract

**Background:**

Despite legislative action, pre-existing barriers continue to prevent patients from using patient portals. Patients, especially older people, people of color, and people with limited English proficiency continue to experience difficulty in adopting patient portals.

**Objective:**

The aim of this study was to advance understanding, explore willingness to adopt an electronic portal, and examine differences between language preferences.

**Methods:**

English- and Spanish-speaking patients (N=106) were surveyed from a community clinic regarding access to electronic devices and the internet, barriers to using a patient portal, willingness to adopt such a portal, preference mode of communication with health care providers, and preferred features in the current clinic’s portal. Linear and logistic regressions were performed to predict the probability that patients would adopt the patient portal.

**Results:**

Only 65% (n=69)of participants said they envisioned themselves using a patient portal. English-speaking patients were more willing to exchange electronic information with their health care providers. Spanish-speaking patients reported language as a significant barrier to portal use. A logistic regression revealed that patients with more positive attitudes and higher perceived behavioral control are more likely to sign up and use the patient portal (Nagelkerke *R*^2^=.51, classification=90.8%, efficacy B=2.38, Wald-1=5.93, *P*=.02 and Exp[B]=12.44, attitude B=1.87, Wald=6.45, *P*=.01, Exp[B]=7.49).

**Conclusions:**

Understanding language preference differences while predicting portal use based on attitudes and perceptions empowers patients to have a more meaningful experience with their physician, potentially overcoming low health literacy–related barriers.

## Introduction

Beginning in 2000, the Office of Civil Rights of the Department of Health and Human Services required physicians to comply with requests for copies of medical records [1]. By 2014, physicians participating in the Electronic Health Record Incentive Program were expected to transition to electronic medical recordkeeping, and finally, the 2021 Cures Act granted patients near immediate access to their medical records and prohibited practices that interfere with the access, exchange, or use of electronic health information [1-6]. Despite legislative action to protect the rights of patients, there remain pre-existing barriers that prevent patients from understanding, signing up for, and using patient portals.

Despite patients reporting generally high access and perceived usefulness attitudes toward patient portals, especially for administrative tasks such as reviewing lab results, patients continue to experience barriers in adopting patient portals [7,8]. Those who use patient portals less frequently tend to be older adults, people of color, adolescents, and people with limited English proficiency (LEP) [9-13]. Previous research has shown that race and ethnicity are the strongest predictors when investigating portal use, especially for Latinx individuals, as reports revealed that people of color and LEP were offered access to patient portals at much lower rates yet are more likely to download and transmit health information, highlighting the need for providers to promote portal use [4,10-12].

In order to provide the best patient portal experience, a multifaceted, multilingual portal platform is necessary. Understanding the limitations of existing clinical electronic systems is critical, as a complete redesign of portals is impractical. Education on current platforms and consideration of language in messages can help alleviate confusion and increase portal uptake [9,12]. Previous studies suggest that patients of color prefer face-to-face interaction with their providers; therefore, it may be beneficial to leverage existing relationships to provide training on the proper use and promotion of existing patient portals [4,11,13,14]. Provider endorsement increases portal use and patient empowerment and self-efficacy [5,8,10,13,15].

Patient portals can help patients interact with their health care team, manage appointments, access medical notes, and have been shown to improve medication adherence and health care quality [4,6,9,16,17]. A 2017 study revealed patient portal use was found to decrease emergency visits and hospital admissions [17]. On some clinical notes sharing platforms, such as Our Notes, patients have the ability to work alongside physicians by contributing their own notes to their digital medical records, resulting in an increase of medication and treatment plan adherence [5,6]. This shared work increases the patients’ perceived ability to control their health, motivating further engagement with patient portals [6,10,15]. In addition, physicians can overcome distrust toward the health care system, mainly felt in patients of color, by increasing communication quality [4,7,14]. Engaging in meaningful conversation also combats the barriers of low health and technology literacy that is largely observed in the older population [6,9,11].

The purpose of this paper was to advance understanding of potential barriers to patient portal use by exploring patients’ willingness to adopt an electronic portal. This understanding is achieved by measuring attitudes and perceived expectations leading to intention and examining differences between distinct linguistic backgrounds, specifically for patients who might experience language and technological barriers. Based on previous research, we hypothesize a presence of distinct differences in barriers to patient portal adoption between clinic patients based on preferred language (English and Spanish). The rest of this paper provides an overview of study methods, results, and a discussion of implications for future research and practice to increase adoption of a potentially important health technology by understanding barriers to use.

## Methods

### Data Collection

This study relied on convenience sampling of patients from a nonprofit Federally Qualified Health Center (FQHC) serving marginalized community members from June 2022 to July 2022. There was no existing user of the patient portal as the study was conducted prior to the full launch to improve the design of the patient portal. The access to the digital survey was published on the clinic’s website and the recruitment material was available at the clinic’s waiting room with a QR code directing to the digital survey. The survey took about 7 minutes to finish. Those who agreed had the chance to opt into a drawing for 20 gift cards (US $25 each). All questions were translated into Spanish by certified translators and double-checked by a native Spanish speaker graduate research assistant.

### Design

The survey asked questions regarding participants’ access to electronic devices and the internet, barriers to using a patient portal, willingness to adopt such a portal, preference mode of communication with health care providers, and preferred features in the current clinic’s portal. To determine the participants' preferred language, participants were asked what their primary language was, and more specifically, what language they speak most of the time. Potential barriers were identified from previous literature as well as input from the clinical staff’s experience [4,6,11,18,19]. For the full survey instrument, please see Multimedia Appendix 1.

Grounded in the Theory of Planned Behavior, a psychological framework focused on behavior prediction, which states intention as the strongest predictor to behavior measured by the attitude toward the behavior, perceived behavioral norms, and perceived behavioral control shown in [20] (Textbox 1). Measures were adopted from previous studies [20-22] and modified to fit the research context of interest and participants’ literacy level per clinic staff’s feedback.

Textbox 1. **Theory of Planned Behavior measures**

**Attitude**

- Using the Patient Portal will support critical aspects of my health care.
- Using the Patient Portal will enhance my effectiveness in managing my health care.
- Overall, the Patient Portal will be useful in managing my health care.

**Perceived behavioral norms**

- People who are important to me think that I should use the Patient Portal.
- People who influence my behavior think that I should use the Patient Portal.
- People whose opinions that I value prefer that I use the Patient Portal.

**Perceived behavioral control**

- Learning how to use the Patient Portal is easy for me.
- My interaction with the Patient Portal is clear and understandable.
- I find the Patient Portal easy to use.It is easy for me to become skillful at using the Patient Portal.

### Data Analysis

A series of independent-sample *t* tests and *χ*^2^ analyses were performed to identify differences among English- and Spanish-speaking participants. A logistic regression analysis was performed to investigate the relationship between attitudes, perceived behavioral control, and perceived behavioral norms with the intention to adopt the patient portal. Age and gender were controlled in the logistic regression.

### Ethical Considerations

The University of Texas at Austin Institutional Review Board determined this study as exempt and did not require further review (STUDY00002291). Participants reviewed and acknowledged informed consent materials prior to completing the electronic survey. To compensate participants, each person was entered into a drawing to win one of the 20 gift cards (US $25 each). For privacy and confidentiality, all study data were deidentified.

## Results

The demographic information of the participants is shown in Table 1. The majority of respondents (96/106, 93%) reported having good internet access, with 91/106 (88%) having access at home and 64/106 (60%) respondents reported owning an internet-enabled device. Specifically, 80 (75%) participants reported accessing the internet at home, 66 (62%) through mobile phones, 24 (22%) at work, 21/106 (20%) via a tablet, 7 out of 106 (7%) at school, 6/106 (6%) public spaces such as a cafe, 6 (6%) from someone else’s home, 4 out of 106 (4%) at a public library, and 1 participant (0.9%) at a community center.

Regarding potential barriers to using an electronic patient portal, 29 (27%) participants reported preferring in-person visits as their main concern, followed by 25 (23%) worry about privacy, 14 (13%) lack of access to the internet, 10 (9%) language difficulties, 7 (7%) lack of comfort with computers and internet-enabled devices, and 3 (3%) participants reported poorly designed or implemented portals.

Specific to this clinic’s patient portal, only 69 (65%) of participants said they saw themselves signing up and using it, and 8 (8%) reported not being sure.

A series of analyses revealed differences among English- and Spanish-speaking participants. An independent-sample *t* test showed that those who speak English were more willing to get electronic information about symptoms than Spanish speakers (*P*=.006). Further, *χ*^2^ analyses showed significant association between language and internet access, communication with doctor preferences, barriers to using a portal, and features that participants preferred about the clinic’s patient portal (Table 2).

**Figure 1.**
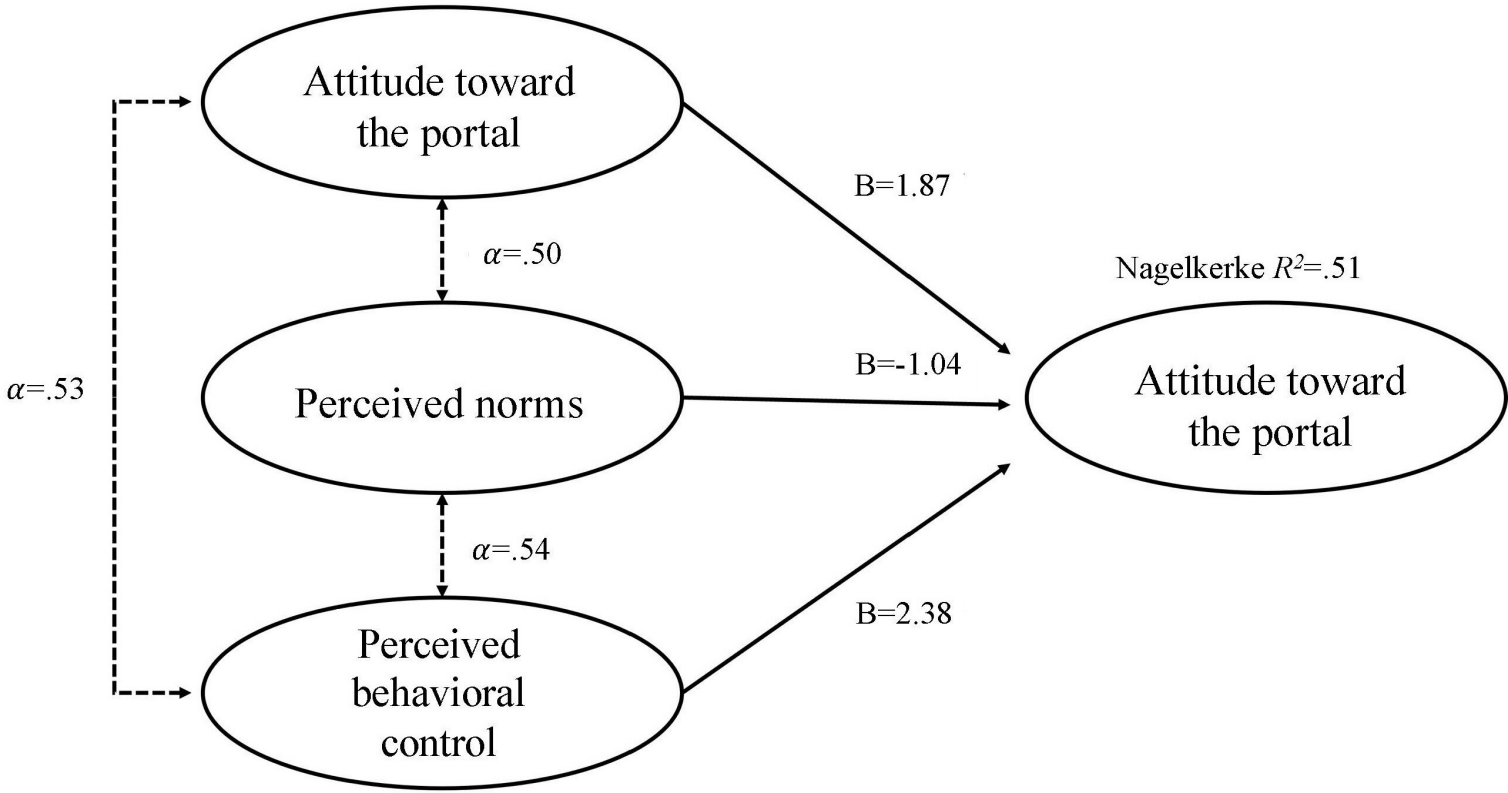
Theory of Planned Behavior correlations.

Based on the Theory of Planned Behavior, Figure 1 shows the results of correlations and the logistic regression. Overall, a logistic regression revealed that patients with more positive attitudes and higher perceived behavioral control are more likely to sign up and use a patient portal (Nagelkerke *R*^2^=.51, classification=90.8%, control B=2.38, odds ratio[OR] 10.81, *P*=.02; attitude B=1.87, OR 6.52, *P*=.02); however, the relationship between perceived norm and sign-up was not significant (norm B=−1.04, OR .35, *P*=.27).

**Table 1.** Baseline demographic details of study population.

Baseline demographics	Participants (N=107)^a^
Sex, n (%)	
Female	90/106 (85)
Male	15/106 (14)
Chose not to disclose	1/106 (1)
Race or ethnicity^b^, n (%)	
African American or Black	16/109 (15)
Asian, Asian Indian, or Asian American	1/109 (1)
American Indian or Alaska Native	3/109 (3)
White	19/109 (17)
Hispanic or Latino or Latina	70/109 (65)
Age (years), mean (SD)	40 (15.35)
Language, n (%)	
English	67/107 (63)
Spanish	40/107 (37)

a a107 participants completed at least 60% of the survey instrument; however, only 106 completed the entire survey instrument.

b Participants were able to select multiple race or ethnicity options.

**Table 2.** Chi-square analyses to examine language associations (N=106).

Variables	Total (N=106), n (%)	English-speaking (n=67), n (%)	Spanish-speaking (n=40), n (%)	*χ*^2^ (df)	*P* value
Internet access and device ownership					
Internet access	96 (93)	64 (100)	32 (82)	12.32^a^	<.001
Internet access at home	91 (88)	62 (95)	29 (74)	9.85^a^	.002
Computer access at home	64 (62)	47 (73)	17 (43)	9.95^a^	.002
Internet use, home	27 (75)	56 (84)	24 (60)	7.38^a^	.007
Internet use, work	24 (22)	20 (30)	4 (10)	5.67^a^	.02
Owning personal computers	48 (45)	40 (60)	8 (20)	15.96^a^	<.001
Communication with doctor preferences					
In-person	63 (59)	34 (51)	29 (73)	4.90^b^	.03
Telephone	52 (49)	41 (61)	11 (28)	11.38^a^	<.001
Email	33 (31)	26 (39)	7 (18)	5.33^a^	.02
Barriers to using an electronic patient portal					
Privacy concerns	25 (23)	22 (33)	3 (8)	8.98^a^	.003
Language	10 (9)	3 (5)	7 (18)	5.01^b^	.03
Most liked portal features					
Paperwork and records	53 (50)	39 (58)	14 (35)	5.40^a^	.02
Asking for medical refills	46 (43)	35 (52)	11 (28)	6.25^a^	.01
Send questions to provider online	44 (41)	36 (54)	8 (20)	11.77^a^	.001

a English-speaking participants were overrepresented.

b Spanish-speaking participants were overrepresented.

## Discussion

### Primary Barriers to Patient Portal Adoption

The differences between English-speaking and Spanish-speaking participants revealed further insights such as Spanish-speaking participants’ lower willingness to exchange electronic information than English-speaking participants. Even so, compared to English-speaking participants, Spanish-speaking participants prefer in-person communication with their physicians. This finding, in conjunction with the language barrier, supports previous research where more emphasis on portal training for patients with LEP is needed to increase comfort and combat distrust [4].

Historically, patient portal uptake has been challenging due to a lack of access to technology [6]. However, the findings of this study revealed many patients have good internet access and device availability. Despite adequate access to the technology, only 65% of participants said they envisioned themselves signing up for and using the patient portal. While this is a fairly strong intention response, there are still barriers worth acknowledging, such as a stronger preference for in-person communication with their physician, concerns about privacy, language difficulties, lack of comfort with portals, and poor design. The most prominent barrier reported was the preference for in-person communication with their doctor, highlighting an opportunity for physicians and staff to emphasize the potential benefit of completing administrative tasks through portal use such as scheduling appointments, sending secure messages, and reviewing lab results.

### Comparison to Prior Work

Many studies have investigated the uptake of patient portals in Hispanic populations; however, often in specialized areas of care such as asthma or with small sample sizes [8,12,19]. Few have investigated patients’ willingness to adopt an electronic portal and assess potential barriers while specifically exploring the impact of language preferences and measuring behavioral intention. This study successfully fills such a gap, as it surveyed English- and Spanish-speaking participants from a community clinic in a Southwestern city to identify patient portal uptake barriers based on language preferences and measured differences in their intention to sign up and use the portal. Understanding the existing barriers is the first step in strategizing ways to overcome obstacles patients experience when considering a patient portal.

### Limitations and Considerations

The Theory of Planned Behavior is criticized for simply measuring the intentions to predict actual behavior; however, this study uniquely addresses this by simultaneously identifying barriers and measuring patients’ attitudes and perceptions (perceived norms and perceived behavioral control) toward portal adoption. The first notable limitation is that while this study did not measure actual adoption of the clinic’s patient portal, the findings still provide insight into the challenges faced by a multilingual patient population. Specifically, by understanding attitudes and perceptions along with language preference differences, we can tailor approaches and predict how patients with higher perceived behavioral control and more positive attitudes toward the patient portal were most likely to adopt and use the portal. The second limitation is the reliance on convenience sampling limiting the ability to make broad generalizations from the findings; however, the strength of this study is that it helps understand the need for further assessment on ways to increase perceived behavioral control and positive attitudes toward patient portals through in-person relationships, particularly among patients with LEP.

### Future Directions

The study’s findings identify areas for health care organizations to focus on when seeking to increase patient portal uptake among a linguistically diverse population. Future research can investigate in-person preferences as well as communication surrounding privacy concerns, which were the top 2 barriers expressed by participants in this study. Empowering patients to have more autonomy over their health and decision-making leads to a more meaningful experience with their physicians, which can tackle low health literacy–related barriers. Yet, understanding these differences based on language preference confirms that a more targeted approach is needed. These findings add to the current body of literature by revealing that barriers such as lack of access require more investigation, as even with adequate access to internet and technology, there is still a need for patients to feel secure with the level of privacy and ease of use regarding portal design.

### Conclusions

To understand the willingness to adopt an electronic portal and examine differences between language preferences, this study presented survey findings from a FQHC regarding access to electronic devices and the internet, and identified barriers to using a patient portal. We conducted a series of independent-sample *t* tests and *χ*^2^ analyses to identify differences among English- and Spanish-speaking participants. A logistic regression analysis was performed to investigate the relationship between attitudes, perceived behavioral control, and perceived behavioral norms with the intention to adopt the patient portal. Despite adequate access to technology, only 65% of participants said they envisioned themselves using a patient portal. English-speaking patients were more willing to exchange electronic information with their health care providers. Spanish-speaking patients reported language as a significant barrier to portal use. Finally, patients with more positive attitudes and higher perceived behavioral control are more likely to sign up and use the patient portal. These findings can inform strategies to achieve a more meaningful experience between physician and patients with potential to tailor approaches to patients based on preferred language.

## Supplementary material

10.2196/66717 Multimedia Appendix 1 Survey instrument.

## Abbreviations

FQHC: Federally Qualified Health Center
LEP: limited English proficiency
OR: odds ratio
