# Supplementary material for: Barriers to Patient Portal Adoption Among a Bilingual Patient Population by Analysis of Survey Findings from English- and Spanish-Speaking Patients: Information Needs Study
Source: JMIR Form Res. 2025 Jul 28;9:e66717. doi: 10.2196/66717 (PMC12303557; doi:10.2196/66717)
Supplement: Multimedia Appendix 1 [file formative-v9-e66717-s001.docx]

**Patient Survey**

**Demographic Information**

1. How old are you today?
2. What is your primary language (i.e., the one you speak most of the time)?
   - English
   - Spanish
   - Other __
3. Which race/ethnicity best describe you? Select all that apply.
   - African American or Black
   - American Indian or Alaska Native
   - Asian, Asian Indian, or Asian American
   - Hispanic, Latino/a/x, or Spanish origin
   - Middle Eastern or North African
   - Native Hawaiian or Pacific Islander
   - White or Caucasian
   - Prefer to self-describe ___
   - Prefer not to answer
4. What is your gender?
   - Male
   - Female
   - Non-binary/Third gender
   - Prefer to self-describe __
   - Prefer not to say

**Internet Access**

1. Do you have stable internet access?
   - Y/N
2. If you answered “Yes” to the question 5, where do you use the Internet? Check all that apply.
   - Home
   - School
   - Work
   - Public library
   - Community center
   - Other public spaces (e.g., a cafe)
   - Someone else’s house
   - Mobile phone
   - Tablet computer
   - Other ___
3. Do you have Internet access at home?
   - Y/N
4. Do you have any type of computer, including laptops, in your home?
   - Y/N
5. Please indicate if you have one of the following electronic devices. Check all that apply.

**Health Information**

1. What resources do you primarily rely upon to manage your health? Select all that apply.
   - Doctor
   - Clinic/Hospital
   - Family
   - Community/Support Groups
   - Internet
   - Other __
2. Outside of an office visit, how do you prefer to communicate with your doctor?
   - In person
   - Telephone Call
   - Letter
   - E-mail
   - Text
   - Other __
3. How willing would you be to exchange the following types of medical information with a health care provider electronically through your computer, laptop, mobile phone or tablet? (Very -- Somewhat -- A little -- Not at all)
   - General health tips
   - Medication reminders
   - Lab test results
   - Diagnostic information (e.g., medical illnesses or diseases)
   - Vital signs (e.g., heart rate, blood pressure, etc.)
   - Lifestyle behaviors (e.g., physical activity, diet, sleep, etc.)
   - Symptoms (e.g., nausea, pain, dizziness, etc.)

**Psychosocial Factors**

1. Please indicate to what extent you agree or disagree with the following statement: I am interested in having online access to my medical records.
   - Strongly disagree
   - Disagree
   - Undecided
   - Agree
   - Strong Agree
2. Please indicate to what extent you agree or disagree with the following statement: Having access to a patient portal, in which you can access your medical record online, improves quality of care.
   - Strongly disagree
   - Disagree
   - Undecided
   - Agree
   - Strong Agree
3. Please indicate to what extent you agree or disagree with the following statement: Having access to a patient portal, in which you can access your medical record online, improves the patient-provider relationship.
   - Strongly disagree
   - Disagree
   - Undecided
   - Agree
   - Strong Agree
4. Which of the following do you believe is the biggest barrier to using an electronic patient portal? Select all that apply.
   - preference for direct in-person communication
   - no internet access
   - privacy concerns
   - not comfortable with computers or smartphones
   - Language barriers
   - Other __
5. Please indicate if you agree or disagree with each of the following statements.

(Agree/Disagree)

- - Using the Patient Portal will support critical aspects of my healthcare.
  - Using the Patient Portal will enhance my effectiveness in managing my healthcare.
  - Overall, the Patient Portal will be useful in managing my healthcare.
  - Learning how to use the Patient Portal is easy for me.
  - My interaction with the Patient Portal is clear and understandable.
  - I find the Patient Portal easy to use.
  - It is easy for me to become skillful at using the Patient Portal.
  - People who are important to me think that I should use the Patient Portal.
  - People who influence my behavior think that I should use the Patient Portal.
  - People whose opinions that I value prefer that I use the Patient Portal.

**PCC Portal Specific Questions**

1. The People’s Community Clinic Patient Portal allows you to have access to the following features. Which features have the most appeal to you? Check all that apply.
   - Secure messaging connects you with your provider, see appointments, receive lab results, and get answers to your medical questions
   - Request, reschedule and cancel appointments at your convenience
   - Pay your provider bills online at your convenience
   - Eliminate phone tag by submitting questions to your provider online
   - Request a refill for your prescription medications
   - Complete paperwork before your appointments and updated your records
2. Can you see yourself using the patient portal?
   - Y/N/Not sure
3. If not, why not? ___
4. If not, what would motivate you to register for the patient portal? ___
5. What questions or concerns do you have about registering for the patient portal? ___

You have the chance to win a $25 gift card (20 gift cards in total)!

If you opt in, please leave your contact information:

Name:

Email:

**Provider Survey**

1. How would you describe your role at People’s Community Clinic? ___
2. Please indicate to what extent you agree or disagree with the following statement: A patient portal is a helpful tool for me.
   - Strongly disagree
   - Disagree
   - Undecided
   - Agree
   - Strong Agree
3. Please indicate to what extent you agree or disagree with the following statement: A patient portal is a helpful tool for a patient.
   - Strongly disagree
   - Disagree
   - Undecided
   - Agree
   - Strong Agree
4. Please indicate to what extent you agree or disagree with the following statement: I would encourage my patients to sign up for and use the PCC patient portal.
   - Strongly disagree
   - Disagree
   - Undecided
   - Agree
   - Strong Agree
5. How would you say a patient portal affects the efficiency of patient care? ___
6. What are barriers to patients using the portal? ___
